# A Competing Risk Nomogram for Predicting Cancer-Specific Death of Patients With Maxillary Sinus Carcinoma

**DOI:** 10.3389/fonc.2021.698955

**Published:** 2021-08-24

**Authors:** Mingbin Hu, Xiancai Li, Weiguo Gu, Jinhong Mei, Dewu Liu, Shaoqing Chen

**Affiliations:** ^1^Department of Oncology, The First Affiliated Hospital of Nanchang University, Nanchang, China; ^2^Department of Burns, The First Affiliated Hospital of Nanchang University, Nanchang, China; ^3^Department of Pathology, The First Affiliated Hospital of Nanchang University, Nanchang, China

**Keywords:** maxillary sinus carcinoma, nomogram, cancer-specific death, SEER, competing risk

## Abstract

**Objectives:**

Herein, we purposed to establish and verify a competing risk nomogram for estimating the risk of cancer-specific death (CSD) in Maxillary Sinus Carcinoma (MSC) patients.

**Methods:**

The data of individuals with MSC used in this study was abstracted from the (SEER) Surveillance, Epidemiology, and End Results data resource as well as from the First Affiliated Hospital of Nanchang University (China). The risk predictors linked to CSD were identified using the CIF (cumulative incidence function) along with the Fine-Gray proportional hazards model on the basis of univariate analysis coupled with multivariate analysis implemented in the R-software. After that, a nomogram was created and verified to estimate the three- and five-year CSD probability.

**Results:**

Overall, 478 individuals with MSC were enrolled from the SEER data resource, with a 3- and 5-year cumulative incidence of CSD after diagnosis of 42.1% and 44.3%, respectively. The Fine-Gray analysis illustrated that age, histological type, N stage, grade, surgery, and T stage were independent predictors linked to CSD in the SEER-training data set (n = 343). These variables were incorporated in the prediction nomogram. The nomogram was well calibrated and it demonstrated a remarkable estimation accuracy in the internal validation data set (n = 135) abstracted from the SEER data resource and the external validation data set (n = 200). The nomograms were well-calibrated and had a good discriminative ability with concordance indexes (c-indexes) of 0.810, 0.761, and 0.755 for the 3- and 5-year prognosis prediction of MSC-specific mortality in the training cohort, internal validation, and external validation cohort, respectively.

**Conclusions:**

The competing risk nomogram constructed herein proved to be an optimal assistant tool for estimating CSD in individuals with MSC.

## Introduction

Maxillary sinus carcinoma (MSC) accounts for 1%–4% of all head and neck cancers (1). Early diagnosis of maxillary sinus is difficult because of its hidden anatomical site and complex adjacent relationship. In most patients it has already invaded the bone wall and surrounding tissues when they are diagnosed, meaning they have a poorly defined prognosis (2). Numerous reports have documented the prognosis of general oral cancer (3) and Nasopharyngeal carcinoma (4), but few have addressed MSC. Numerous reports have documented the survival of individuals with MSC. Nonetheless, most reports are based on single medical institutions, with small sample sizes (5). Therefore, it is critical to strengthen research on MSC prognosis.

Surveillance, Epidemiology, and End Results (SEER), a population-based data resource, has data for about 28% of the US population. Hence, particularly for rare tumors, there are a number of relevant cases in the SEER data resource that can be used for establishing competitive risk prediction models (6). The information of MSC cases herein was abstracted from the SEER data resource, which guarantees the sufficiency, as well as authenticity of the data. Generally, patients with cancer are often predisposed to more than two risks, however only one event finally occurs (7). The risks other than the one of interest are referred to as competing risks. Competing risks are censored in a traditional survival analysis, but can be improved *via* a competing risk analysis.

A nomogram is a visualization of a linear prognostic model that is employed to quickly predict survival probabilities (8). Each value of these characteristics reflects a score on the nomogram graph, with the overall score mapping the survival likelihood. Some researches only focus on the traditional Kaplan-Meier method along with Cox proportional hazard model, while some studies are centered on population-based assessments (9, 10). Nevertheless, a remarkable amount of research has explored the overall survival along with cancer cause-distinct survival analysis, neglecting the involvement of competing causes of death in non-metastatic MSC prognosis. The competing risks of death influence the long-time survival prognosis to a remarkable extent; therefore, they should be taken into account when predicting the survival outcomes.

Herein, we aimed to develop a competing risk nomogram on the basis of the data abstracted from the SEER data resource for estimating cancer-specific death (CSD) in individuals with MSC. This could help clinicians in making decisions regarding individualized MSC treatment, as well as making accurate estimations of disease outcomes.

## Material And Methods

### Surveillance, Epidemiology, and End Results Database Patients

This was a retrospective analysis that analyzed the data of individuals with MSC between 2000 and 2017. The data of the individuals with MSC used in this study were abstracted from the publicly accessible SEER data resource.

SEER 18 Regs custom data (with additional treatment fields) uploaded in November 2019 (1975–2017 varying) were selected. All individuals diagnosed with MSC (site recode NM7/CS v0204+ Schema of “Sinus Maxillary” and behavior recode ICD-O-3 of “malignant”) were enrolled. Participants who were less than 5 years old at diagnosis, with a survival period of less than or equal to one month lacking a pathological diagnosis or lacking complete data were excluded from the study.

### Our Medical Center Patients

Overall, we enrolled 200 individuals with MSC from the First Affiliated Hospital of Nanchang University (China) from 2006 to 2017. All patients were confirmed by pathology, and had no history of other cancers. The approval of this study was granted by the Ethics Committee of First Affiliated Hospital of Nanchang University (No. 2020140).

### Variable Selection

The variables consisting of Age, Race, Sex, AJCC (American Joint Committee on Cancer) stage, T stage, N stage, M stage, Surgery, Grade, Radiation, histological type, follow-up time as well as survival outcomes were abstracted from the SEER data resource. The X-tile software (https://x-tile.software.informer.com/) was employed to explore the optimal cut-point values. The age at the time of diagnosis of the patients was categorized into two groups, i.e., <65 and ≥65 years. The AJCC staging approach, seventh edition was utilized herein. The ICD-O-3 codes was employed to stratify the MSC histological type into two classes, i.e., SCC (squamous cell carcinoma) and none SCC (consisting of adenomas and adenocarcinomas, cystic, adnexal and skin appendage neoplasms, mucinous, serous neoplasms, and mucoepidermoid neoplasms, etc.) on the basis of the WHO classification approach. Tumor-specific survival was the primary endpoint in this study, which was computed as the time from MSC diagnosis to the death of the participant resulting from MSC or a censored event. Deaths resulting from accidents or diseases apart from MSC were regarded as competitive risks.

### Statistical Analyses

All analyses were implemented in the R-software (V.4.0.4; packages: foreign, cmprsk, mstate, rms, crrstep, pec, survivial, and riskRegression). P-value was two-sided, p < 0.05 defining statistical significance. First, we determined the CIF (cumulative incidence function) for 3- to 5-year time points. Additional subgroup analyses were carried out between various subgroups, and respective CIF curves were constructed for these variables. Remarkable differences in the CIF values among subgroups were explored with the Gray’s test. Secondly, we randomly split the enrolled SEER data resource participants into a training data set and a validation data set at a ratio of 7:3. The external validation data set consisted of subjects with MSC enrolled from our hospital. The training data set was utilized to construct the nomogram that was employed to estimate CSD. The two validation data sets were used in verifying the accuracy of the constructed nomogram. Univariate along with multivariate analyses were utilized to determine the independent risk factors of CSD in the training data set. The Fine-Gray proportional hazards model was employed to construct the competing risk nomogram.

The nomogram efficiency was first assessed in the training data set and then in the validation data sets regarding the C-index, AUC, as well as calibration curve. The C-index was employed to quantify the estimation potential of the model. It ranged between 0.5 and 1.0, which reflected a random chance from revealing no discrimination to revealing perfect discrimination (11). The AUC exhibits the overall prognostic value across all thresholds (12), with an optimum estimation value yielding an AUC of 1.0. DCA (decision curve analysis) was employed to establish the clinical net benefit of diverse prognostic thresholds for a prospective clinical effect (13), and evaluated the nomogram performance in comparison with the AJCC staging system visually.

## Results

### Baseline Characteristics

**Figure 1:**
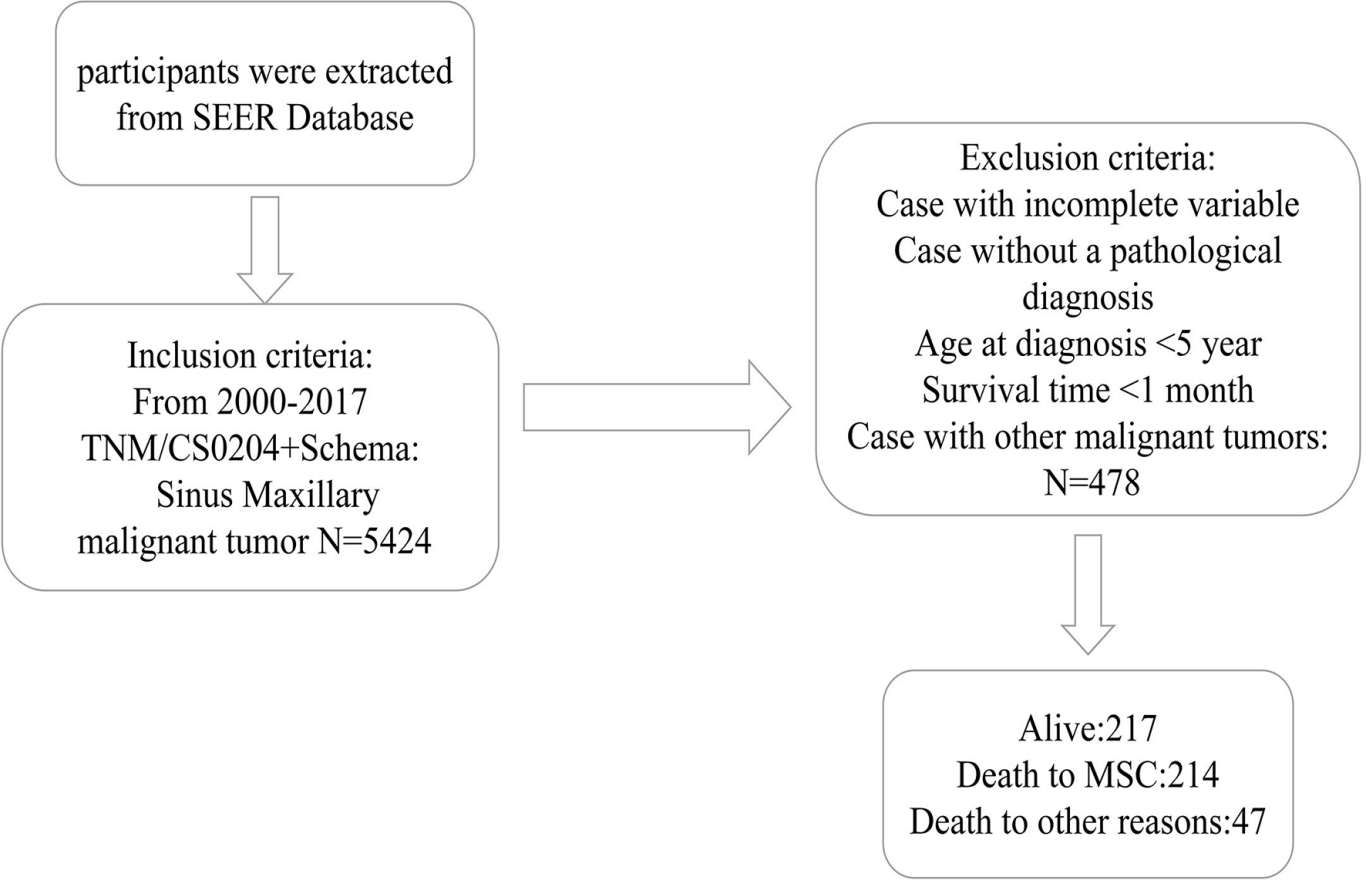
The Flow diagram of patient selection. SEER, Surveillance, Epidemiology, and End Results; MSC, Maxillary Sinus Carcinoma.

As indicated in **Figure 1**, initially, the data of 5,424 individuals with MSC was abstracted from the SEER data resource. Following the thorough screening, 478 individuals with MSC were enrolled in the final analysis. The median age of the patients was 64 (15–85) years at diagnosis (males = 66.7%). Most of the patients were of the white race (*n *= 345, 72.2%). Of the 478 MSC cases, 264 (55.2%) were SCC, including 202 (42.3%) cases of moderate differentiation. Stage IV was the most prevalent tumor stage (*n *= 176, 36.8%), followed by stage III (*n *= 171, 35.8%), II (*n *= 95, 19.9%), and I (*n* = 36, 7.5%). A remarkable number of the patients were classified as T4 (59.4%), followed by T3 (22.2%), T2 (10.7%), and T1 (7.7%). More than 50% of the cases were without lymph node (LN) metastasis (N0, 77.8%), and most cases had no distant metastasis (M0, 95.0%). A remarkable number of the patients were treated with surgery (*n *= 346, 72.4%). According to the observation of clinical characteristics in the three cohorts, there were differences in age, AJCC stage, T stage, N stage, surgery, radiation, and histologic type (P < 0.05). **Table 1** provides a detailed summary of the demographic along with the clinical features of the enrolled participants.

**Table 1:** Basic characteristics of Maxillary Sinus Carcinoma patients in the training, internal validation, and external validation cohorts.

Characteristics	SEER database		Our medical center	p-value
	Training cohort (n = 343) n (%)	Internal validation cohort (n = 135) n (%)	External validation cohort (n = 200) n (%)	
Age(years)				<0.001
<65	219 (63.8)	100 (74.1)	166 (83.0)	
≥65	124 (36.2)	35 (25.9)	34 (17.0)	
Race				0.247
White	246 (71.7)	99 (73.3)		
Black	56 (16.3)	20 (14.8)		
Others	41 (12.0)	16 (11.9)	200 (100)	
Sex				0.966
Male	234 (68.2)	93 (68.9)	78 (39.0)	
Female	109 (31.8)	42 (31.1)	122 (61.0)	
AJCC stage				<0.001
I	21 (6.1)	15 (11.1)	14 (7.0)	
II	81 (23.6)	14 (10.4)	49 (24.5)	
III	121 (35.3)	50 (37.0)	101 (50.5)	
IV	120 (35.0)	56 (41.5)	36 (18.0)	
T stage				<0.001
T1	29 (8.5)	8 (6.0)	16 (8.0)	
T2	34 (9.9)	17 (12.5)	69 (34.5)	
T3	81 (23.6)	25 (18.5)	83 (41.5)	
T4	199 (58.0)	85 (63.0)	32 (16.0)	
N stage				<0.001
N0	265 (77.3)	107 (79.3)	114 (57.0)	
N1	30 (9.2)	7 (5.2)	71 (35.5)	
N2	48 (13.5)	21 (15.5)	15 (7.5)	
M stage				0.7098
M0	324 (94.5)	130 (96.3)	190 (95.0)	
M1	19 (5.5)	5(3.7)	10 (5.0)	
Surgery				<0.001
No	101 (29.4)	31 (23.0)	11 (5.5)	
Yes	242 (70.6)	104 (77.0)	189 (94.5)	
Grade				<0.001
Well	36 (11.1)	28(20.7)	60 (30.0)	
Moderate	160 (46.6)	42 (31.1)	26 (13.0)	
Poorly/Undifferentiated	147 (42.3)	65 (48.2)	114 (57.0)	
Radiation				<0.001
No	138 (40.2)	49 (36.3)	48 (24.0)	
Yes	205 (60.8)	86 (64.7)	152 (76.0)	
Histologic type				<0.001
SCC	190 (55.5)	74 (54.8)	135 (67.5)	
None SCC	153 (45.5)	61 (45.2)	65 (32.5)	

AJCC, American Joint Committee on Cancer; SCC, squamous cell carcinoma.

### Cumulative Incidence Function Survival Analysis

**Figure 2:**
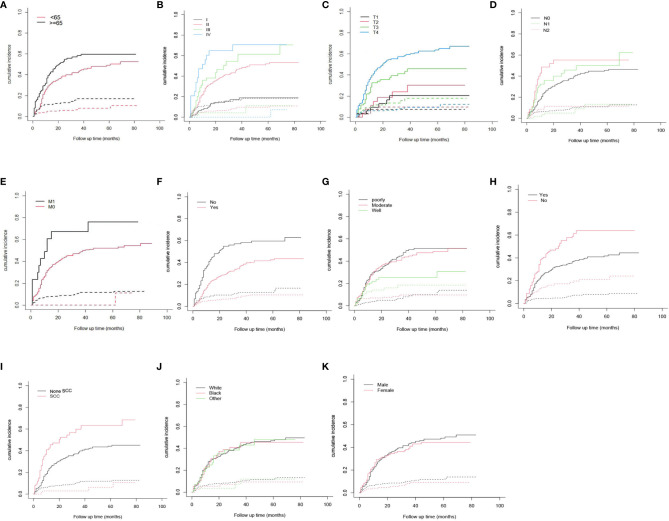
Cumulative incidence estimates of cancer-specific death in Maxillary Sinus Carcinoma. **(A)** Age; **(B)** AJCC stage; **(C)** T stage; **(D)** N stage; **(E)** M stage; **(F)** Surgery; **(G)** Grade; **(H)** radiation; **(I)** Histologic type; **(J)** Race; **(K)** Sex. Solid line represents cause-specific death, dotted line represents other causes of death. AJCC, American Joint Committee on Cancer; SCC, squamous cell carcinoma.

**Table 2** illustrates the results of our competing risk model. The median follow-up period was 26 (1–83) months. Overall, there were 261 deaths (54.6%) by the end of follow-up, with 214 (82.0%) being CSDs and 47 (18.0%) caused by other events. The 3-year cumulative incidence of CSD was 42.1%, while that of the 5-year was 44.3%. The result of the CIF subgroup analysis illustrated that a high CSD primarily occurred in patients aged ≥65 years (**Figure 2A**); who had an advanced AJCC stage (**Figure 2B**), T stage (**Figure 2C**), N stage (**Figure 2D**), and M1 stage (**Figure 2E**); who were not treated with surgery (**Figure 2F**) nor radiation (**Figure 2H**); who had a poorly/undifferentiated grade (**Figure 2G**); and SCC (**Figure 2I**). Nonetheless, no remarkable difference in CSD was observed in race, as well as sex subgroup analyses (**Figures 2J, K** **)**.

**Table 2:** Cumulative incidence of cancer-specific death in Maxillary Sinus Carcinoma.

Characteristics	Total number of pateints (n)	Cumulative incidence		P-value
		3-year	5-year
Age(years)				<0.001
<65	319	43.3%	54%	
≥65	159	56%	56.2%	
Race				0.611
White	345	46.7%	49%	
Black	76	53.5%	53.5%	
Others	57	42.9%	47.9%	
Sex				0.742
Male	327	48.4%	67.7%	
Female	151	45.8%	47,0%	
AJCC stage				<0.001
I	36	18.9%	18.9%	
II	95	49.7%	53%	
III	171	57%	77%	
IV	176	64%	70%	
T stage				<0.001
T1	37	15.6%	16.8%	
T2	51	33.2%	35.3%	
T3	106	55.6%	58.3%	
T4	284	57.7%	61.2%	
N stage				<0.001
N0	372	19.3%	21.6%	
N1	37	47.3%	49.4%	
N2	69	60.3%	60.9%	
M stage				<0.001
M0	454	32.5%	34.6%	
M1	24	82.6%	87.0%	
Surgery				<0.001
No	132	63.1%	64.2%	
Yes	346	41.6%	44.2%	
Grade				<0.001
Well	64	28%	29.7%	
Moderate	202	47.2%	51%	
Poorly/Undifferentiated	212	51.2%	52.5%	
Radiation				<0.001
No	187	63.9%	63.9%	
Yes	291	38.6%	41.6%	
Histology type				<0.001
SCC	264	35.5%	38.9%	
None SCC	214	26.0%	27.5%	

AJCC, American Joint Committee on Cancer; SCC, squamous cell carcinoma.

### Nomogram Construction

**Figure 3:**
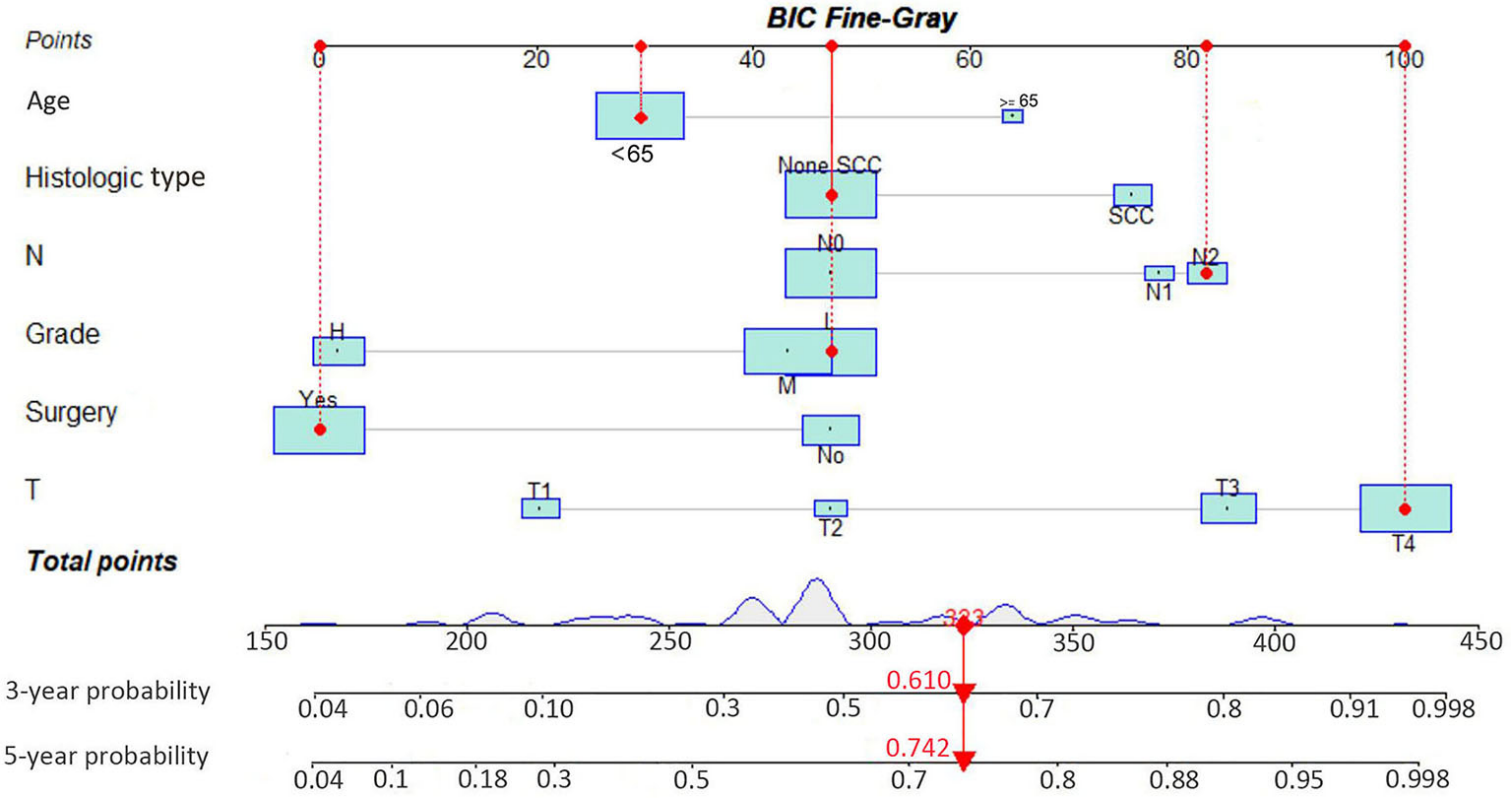
Interactive nomogram for predicting the 3- and 5-year probabilities of cancer-specific death in Maxillary Sinus Carcinoma. BIC, Bayesian information criterion; SCC, squamous cell carcinoma.

As illustrated in **Table 2**, the individuals with MSC abstracted from the SEER database were randomized into a training data set (*n *= 343) and a validation data set (*n *= 135) at a ratio of 7:3. The multivariate analysis of the Fine-Gray proportional sub-distribution hazards model on the basis of the Akaike information criterion (AIC) indicated that age, histological type, stage, grade, N stage, M stage, and surgery were independent predictors affecting CSD in MSC patients of the training group (P < 0.05). After the optimization of the model on the basis of Bayesian information criterion (BIC), six variables were finally included in the prediction model (**Table 3**). A competing risk nomogram was constructed to estimate the 3- and 5-year likelihoods of CSD on the basis of these predictors (**Figure 3**). An individual patient chance of death from MSC at diverse time points could be easily calculated through this prediction model *via* adding the scores of each incorporated variable.

**Table 3:** Results of univariate and multivariate analyses by Fine-Gray proportional sub-distribution hazards model in the training cohort.

Characteristics	Univariate analysis		Multivariate analysis (AIC)		Multivariate analysis (BIC)	
	HR(95% CI)	P-value	HR(95% CI)	P-value	HR(95% CI)	P-value
Age(years)						
<65	Ref		Ref		Ref	
≥65	1.610 (1.300–1.980)	<0.001	1.444(1.140–1.830)	0.002	1.438 (1.136–1.821)	0.003
Race						
White	Ref					
Black	1.070 (0.746–1.550)	0.700				
Others	0.080 (0.588–1.090)	0.160				
Sex						
Male	Ref					
Female	1.110 (0.89–1.370)	0.340				
AJCC stage						
I	Ref					
II	1.670 (1.140–2.450)	0.009				
III	3.130 (2.150–4.540)	<0.001				
IV	6.580 (4.810–9.010)	<0.001				
T stage						
T1	Ref		Ref		Ref	
T2	2.020 (1.520–2.690)	<0.001	1.284 (0.951–1.734)	0.100	1.296 (0.960–1.751)	0.090
T3	4.010 (2.810–5.740)	<0.001	2.244 (1.545–3.258)	<0.001	2.215 (1.514–3.240)	<0.001
T4	4.710 (3.480–6.380)	<0.001	1.808 (1.253–2.610)	<0.001	1.937 (1.353–2.775)	<0.001
N stage						
N0	Ref		Ref		Ref	
N1	2.690 (2.020–3.580)	<0.001	1.848 (1.353–2.525)	<0.001	1.939 (1.426–2.638)	<0.001
N2	4.640 (3.650–5.890)	<0.001	2.981 (2.235–3.978)	<0.001	3.217 (2.437–4.247)	<0.001
M stage						
M0	Ref		Ref			
M1	5.460 (3.640–8.180)	<0.001	2.028 (1.087–3.783)	0.026		
Surgery						
No	Ref		Ref		Ref	
Yes	0.254 (0.200–0.322)	<0.001	0.462 (0.342–0.623)	<0.001	0.451 (0.336–0.606)	<0.001
Radiation						
No	Ref					
Yes	1.54 (1.00–2.325)	<0.001				
Grade						
Well	Ref		Ref			
Moderately	1.510 (1.160–1.960)	<0.001	1.058 (0.803–1.394)	0.847	1.023 (0.723–1.425)	0.423
Poorly/Undifferentiated	2.440 (1.790–3.330)	<0.001	1.495 (1.050–2.129)	0.026	1.235 (1.058–1.919)	<0.001
Histologic type						
SCC	Ref		Ref		Ref	
None SCC	0.169 (0.096–0.296)	<0.001	0.312 (0.176–0.552)	<0.001	0.314 (0.152–0.616)	<0.001

AJCC, American Joint Committee on Cancer; SCC, squamous cell carcinoma.

### Nomogram Verification

**Figure 4:**
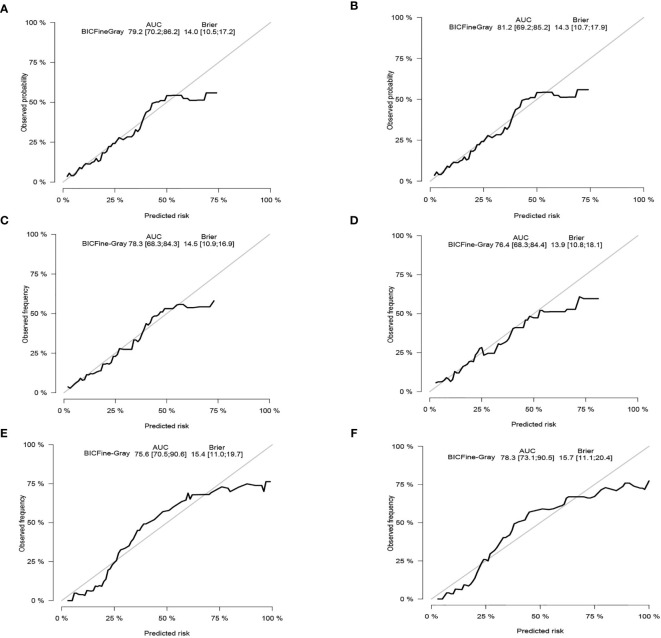
Calibration curves. In the training cohort, the 3-and 5-year probabilities of cancer-specific death **(A, B)**. In the internal validation cohort, the 3- and 5-year probabilities of cancer-specific death **(C, D)**. In the external validation cohort, the 3- and 5-year probabilities of cancer-specific death **(E, F)**. BIC, Bayesian information criterion; AUC, area under the curve.

The C-index of the competing risk nomogram model for estimating the probability of CSD was 0.810 in the training cohort, 0.761 in the internal validation data set, and 0.755 in the external validation cohort. The AUC of our nomogram model for estimating the 3- and 5-year likelihoods of CSD was 0.792 and 0.812 in the training data set, 0.783 and 0.764 in the internal validation data set, and 0.756 and 0.783 in the external validation data set. The calibration graphs exhibited an excellent agreement between the actual and the nomogram-estimated likelihoods in the training (**Figures 4A, B**) and validation data sets (**Figures 4C–F**). Altogether, these data demonstrated the excellent estimation potential along with the remarkable confidence of the constructed nomogram.

### Decision Curve Analysis

**Figure 5:**
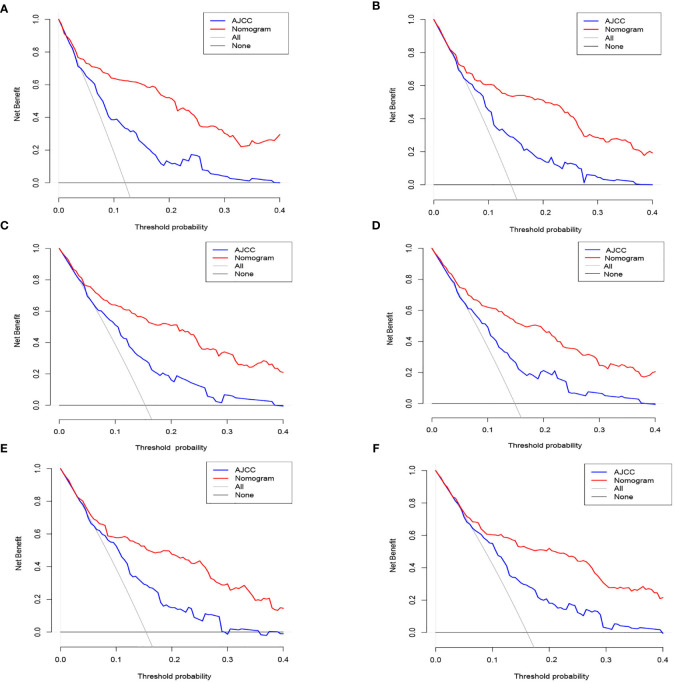
The nomogram of the Decision curve analysis and the nomogram of the AJCC staging system in the prediction of the cancer-specific death of patients at the 3- and 5-year point in the training **(A, B)**, internal validation **(C, D)** and external validation **(E, F)** cohorts.

DCA was conducted in the three study data sets. In all three cohorts, the nomogram illustrated a higher net benefit along with a wider range of threshold likelihood relative to the AJCC staging approach, which depicts that the nomogram showed a high clinical utility value (**Figures 5A–F**).

## Discussion

Maxillary sinus carcinoma is one of the most frequent malignant tumors in the department of otolaryngology. The incidence of MSC is second only to nasopharyngeal carcinoma and laryngeal carcinoma in China, accounting for 2%–3% of head and neck tumors. Previous studies on sinonasal malignancies using the data abstracted from the SEER data resource have focused on incidence, as well as survival patterns (14, 15). For the first time, herein, we constructed prognostic models for the prognosis of individuals with MSC in a competitive event model and established more accurate predictors. The large data samples abstracted from the SEER data resource reduced the error of this study. In contrast with the traditional survival analysis, the competitive event model ensures that the chosen influencing factors have the most direct association with the prognosis of cancer. Although the AJCC staging system is a widely used system at present, it is unable to make a more personalized evaluation on the patient prognosis. For instance, the AJCC TNM staging approach for cutaneous melanoma was suggested to be used in vulva melanoma (16); however, treatment choices, for instance, chemotherapy, surgery, or radiotherapy, were not incorporated in this staging tool. Instead, a nomogram can make a more comprehensive and personalized evaluation because it integrates multiple factors.

Of the 11 parameters discovered herein, nine (age, M stage, radiation, AJCC stage, grade, T stage, surgery, N stage, as well as histological type) were demonstrated to be independent predictors of CSD in individuals with MSC through univariate along with multivariate competing risk analysis. In the univariate analysis, sex and race were not included, implying that they have no impact on CSD in individuals with MSC. The multivariate competing risk analysis data demonstrated that AJCC stage is not an independent predictor, which is linked to its comprehensive assessment of the T, N, and M stages. Following BIC optimization, six parameters (age, histological type, N stage, grade, surgery, and T stage) were included in the model.

It is critical to note that age was found to be an independent factor, which is consistent with Shen et al., who established a nomogram to study the prognosis of MSC. Le et al. explored the staging of MSCs and illustrated that the age of patients, favoring the young, is a remarkable independent predictor after correcting for other confounders, which may be a result of older patients having more comorbidities, as well as higher perioperative risks (15, 17, 18). The result about race in our study is similar with Shen et al., who reported that there was no significant difference in the prognosis among different races (15). There is also no direct evidence of survival differences between races. The research of Wang et al. illustrated that sex had no influence on cancer-specificity survival of Maxillary sinus SCC patients, which is consistent with our research (5). The data illustrated that higher pathological stage (grade) along with M stage and radiation were independent predictors for individuals with MSC, which is congruent with the data of previous studies (19). Nonetheless, the three factors above were removed in the process of using MSC to optimize the model to avoid overfitting.

Most clinicians prefer surgical therapy for MSC at all stages, although on the basis of the NCCN guidelines, surgery is remarkably recommended as the preferred approach for a resectable Maxillary sinus squamous cell carcinoma (MSSCC) (T1–T4a) (20). Our data illustrated that treatment with surgery remarkably reduced the CSD risks in individuals with MSC, which is congruent with the clinical experience of most doctors. However, whether a clinically negative neck in patients with MSC should be treated with an elective neck dissection or irradiated prophylactically is controversial in Europe and the United States (21, 22). In the study of Shen et al., surgery improved survival on the basis of the log-rank test. Nevertheless, in the Cox model, they demonstrated that this protective influence applies only to individuals with negative lymph nodes (15).

MSSCC is the most frequent pathological type in MSC, responsible for about 30%–50% of malignant paranasal sinus tumors (23, 24). Studies have avoided making a comparative analysis between SCC and other kinds of oral cancer. Herein, SCC was responsible for 55.2% of all MSC cases, and we established that the risk of CSD in individuals with SCC was remarkably higher in contrast with that in other kinds of MSC, including adenoid cystic carcinoma, adenocarcinomas, mucoepidermoid neoplasms, and neuroendocrine carcinoma. This is congruent with the findings of Unsal et al., and van der Laan et al. in lung cancer (19, 25). This could be attributed to the high invasive, as well as metastatic ability of squamous cell carcinoma.

Previous reports on sinonasal malignancies that used the SEER program data have focused on incidence along with survival patterns (24, 26), while we centered on constructing estimation models herein. The treatment of MSC and the assessment of the prognosis presently depend on the AJCC staging approach. Our predictive model is appropriate for all individuals with MSC and could be broadly utilized at all levels of medical centers. The comprehensiveness of this nomogram could compensate the inefficiencies of the AJCC staging tool, and allow a precise assessment of the prognosis of individuals with MSC. In addition, a user-friendly graphic interface of the prediction model could enhance communication between clinicians and patients. Besides, we employed a validation data set for external verification, and the results were remarkably linked to the actual survival probabilities.

It is undeniable that this study has some limitations. Firstly, the SEER data resource lacks some critical variables linked to prognosis, such as chemotherapy, perineural infiltration, and smoking and sinusitis history. In addition, we used the sixth or seventh edition of the AJCC staging approach herein, which lacks two critical variables (depth of infiltration and extranodal extension) relative to the eighth edition. Third, the SEER data resource additionally does not collect data on tumor volume, which is regarded as a remarkable prognostic variable for sinonasal malignancies. Although this study included data on radiotherapy, the SEER data resource lacks detailed information on the clinical treatment. Finally, although the SEER data resource provided a large sample size for this study, there are still some errors when it is applied in a global context. Larger-sample multi-center prospective research is required to further improve our prediction model and verify its clinical utility value.

## Conclusion

We have established a competing risk analysis nomogram for individuals with MSC using the data abstracted from the SEER data resource. Our well-calibrated nomogram could be employed to make clinical decisions with regard to the prognosis and individualized treatment of individuals with MSC.

## Data Availability Statement

The data of MSC patients searched in SEER database are freely available. The data collected from our hospital generated and analyzed during the current study are available from the corresponding author on reasonable request.

## Ethics Statement

Written informed consent was obtained from the individual(s) for the publication of any potentially identifiable images or data included in this article.

## Author Contributions

WG and MH were involved in the collecting of data and follow-up of the patients. XL and MH were responsible for the conception and design of the study, assisted with the statistical analysis, and wrote and revised the manuscript. SC, DL, and JM contributed their help on the data analysis, revised the English language and grammar, and corrected parts of the discussion. All authors contributed to the article and approved the submitted version.

## Funding

This work was supported by the National Natural Science Foundation of China (NO.81560410) and Postgraduate Innovation Special Foundation of Jiangxi Province (YC2020-B043).

## Conflict of Interest

The authors declare that the research was conducted in the absence of any commercial or financial relationships that could be construed as a potential conflict of interest.

## Publisher’s Note

All claims expressed in this article are solely those of the authors and do not necessarily represent those of their affiliated organizations, or those of the publisher, the editors and the reviewers. Any product that may be evaluated in this article, or claim that may be made by its manufacturer, is not guaranteed or endorsed by the publisher.
